# Consistent Long-Term Therapeutic Efficacy of Human Umbilical Cord Matrix-Derived Mesenchymal Stromal Cells After Myocardial Infarction Despite Individual Differences and Transient Engraftment

**DOI:** 10.3389/fcell.2021.624601

**Published:** 2021-02-04

**Authors:** Tiago L. Laundos, Francisco Vasques-Nóvoa, Rita N. Gomes, Vasco Sampaio-Pinto, Pedro Cruz, Hélder Cruz, Jorge M. Santos, Rita N. Barcia, Perpétua Pinto-do-Ó, Diana S. Nascimento

**Affiliations:** ^1^Instituto de Investigação e Inovação em Saúde (i3S), University of Porto, Porto, Portugal; ^2^Instituto Nacional de Engenharia Biomédica (INEB), University of Porto, Porto, Portugal; ^3^Instituto de Ciências Biomédicas Abel Salazar (ICBAS), University of Porto, Porto, Portugal; ^4^Cardiovascular RandD Center, Faculty of Medicine of the University of Porto, Porto, Portugal; ^5^Department of Internal Medicine, Centro Hospitalar Universitário São João, Porto, Portugal; ^6^ECBio S.A., Amadora, Portugal

**Keywords:** mesenchymal stromal (or stem) cells, Wharton's jelly, myocardial infarction, regeneration/repair, umbilical cord matrix derived mesenchymal stromal cells (hUCM-MSCs), cell therapy, donor variability, cardiac fibrosis

## Abstract

Human mesenchymal stem cells gather special interest as a universal and feasible add-on therapy for myocardial infarction (MI). In particular, human umbilical cord matrix-derived mesenchymal stromal cells (UCM-MSC) are advantageous since can be easily obtained and display high expansion potential. Using isolation protocols compliant with cell therapy, we previously showed UCM-MSC preserved cardiac function and attenuated remodeling 2 weeks after MI. In this study, UCM-MSC from two umbilical cords, UC-A and UC-B, were transplanted in a murine MI model to investigate consistency and durability of the therapeutic benefits. Both cellular products improved cardiac function and limited adverse cardiac remodeling 12 weeks post-ischemic injury, supporting sustained and long-term beneficial therapeutic effect. Donor associated variability was found in the modulation of cardiac remodeling and activation of the Akt-mTOR-GSK3β survival pathway. *In vitro*, the two cell products displayed similar ability to induce the formation of vessel-like structures and comparable transcriptome in normoxia and hypoxia, apart from UCM-MSCs proliferation and expression differences in a small subset of genes associated with MHC Class I. These findings support that UCM-MSC are strong candidates to assist the treatment of MI whilst calling for the discussion on methodologies to characterize and select best performing UCM-MSC before clinical application.

## Introduction

Cardiovascular diseases are the leading cause of morbidity and mortality worldwide (Benjamin et al., 2019), with ischemic heart disease representing the largest single cause of death in countries of all income levels (Nowbar et al., 2019). Myocardial Infarction (MI) occurs upon blockage of coronary arteries and impaired regional blood supply to the myocardium. As result of nutrient and oxygen deprivation, extensive cardiomyocyte death triggers a sequence of key inflammatory and fibrotic mechanisms that, coupled with the limited regenerative capacity of the adult heart (Sampaio-Pinto et al., 2020), lead to the formation of a non-functional collagen-based scar that negatively impacts on myocardial function and contribute to the development of heart failure. Despite tremendous improvement in the treatment and prognosis of MI, achieved with early reperfusion and optimized pharmacological therapy, some patients with severe and diffuse coronary disease still experience significant ventricular remodeling, myocardial dysfunction and high morbidity and mortality.

Cardiac cell-based therapies aimed at regeneration and/or instructing a more favorable repair have been explored in clinical settings, including skeletal myoblasts, embryonic stem cells (ESCs), bone marrow mononuclear cells (BMMNCs), cardiac stem cells (CSCs), hematopoietic stem cells (HSCs), mesenchymal stromal cells (MSCs), and recently, induced pluripotent stem cells (iPSCs)-derived cardiomyocytes in pre-clinical studies (Madigan and Atoui, 2018). The existence of CSCs in the adult myocardium has raised controversy in particular what concerns their capacity to generate cardiomyocytes (Valente et al., 2014; Maliken and Molkentin, 2018), as their therapeutic effect has been associated to immunomodulatory and paracrine mechanisms also observed in human MSCs (Wagner et al., 2020). Indeed, envisioning the development of a universal and feasible therapy, MSCs are of particular interest (Ballini et al., 2017). Contrarily to other candidates for cell therapy, MSCs do not express MHC Class II and display low levels of MHC Class I proteins, as such seen as immune evasive (Ankrum et al., 2014) and thus suitable for MHC mismatched allogeneic transplantation. These cells can be procured from a variety of adult sources as the bone marrow and adipose tissue, and neonatal sources, including the placenta, umbilical cord blood or umbilical cord matrix (UCM; Wharton's Jelly). Among these, UCM-MSC are particularly attractive since the source tissue can be obtained in a non-invasive and more efficient fashion, have higher expansion potential, higher differentiation range and were shown to be stronger immunomodulators by repressing T-cell activation and promoting Treg expansion more efficiently (Santos et al., 2013).

Our previous work showed that human MSCs isolated from the UCM [obtained using proprietary technology (UCX®)] preserved cardiac function and attenuated cardiac remodeling 2 weeks after MI through paracrine mechanisms (Nascimento et al., 2014). To date, one phase 1/2 clinical trial was completed to evaluate safety and efficacy of UCM-MCSs specifically in the treatment of acute MI with ST elevation (Gao et al., 2015), followed by two others directed to heart failure with reduced ejection fraction (Zhao et al., 2015; Bartolucci et al., 2017). While intravascular delivery of UCM-MSC appears safe and leads to improvement of heart function and other clinical indicators (Thompson et al., 2020), little is known regarding the extent of engraftment and whether donor-to-donor variability may influence the therapeutic potency of these cellular products or their derivatives, e.g., conditioned media and extracellular vesicles. Moreover, donor variability is a concern transversal to all MSCs based therapies, independently of tissue sources, as it could potentially lead to confounding effects when only one donor is selected to represent an experimental group. Studies with multiple donors' functional assays *in vivo* have highlighted this issue, with reports of umbilical cord blood (UCB)-MSCs donor variability in response to hypoxic preconditioning and amelioration of limb ischemia (Kang et al., 2018), as well as in a rat model of MI in which therapeutic efficacy was positively correlated with n-cadherin expression (Lee et al., 2012). In the latter work, cell-cell contact mediated by n-cadherin induced activation of ERK and upregulation of VEGF as shown by overexpression and blocking approaches (Lee et al., 2012).

In this study, UCM-MSC from two umbilical cords were isolated and their therapeutic efficacy after MI was compared to evaluate consistency and long-term effect. Intramyocardial delivery of both cellular products in a murine MI model attenuated cardiac dysfunction and minimized adverse cardiac remodeling 12 weeks post ischemic injury, supporting a sustained and long-term beneficial therapeutic effect for this cell product. Despite this beneficial effect, donor associated variability in the modulation of cardiac remodeling and activation of survival pathways was evident. *In vitro*, the two cell products showed equal ability to boost the formation of vessel-like structures and a similar transcriptome in normoxia and hypoxia, apart from expression differences in a small subset of genes associated with MHC Class I. These findings support that UCM-MSC are a strong candidate as add-on therapy for MI whilst calling for the discussion on methodologies to characterize and select best performing UCM-MSC before application in cellular therapies, or alternatives to overcome this limitation.

## Methods

### Ethics and Regulation—Umbilical Cord Samples

This study was performed in accordance with the Declaration of Helsinki and approved by the Ethics Committee at the Cascais Hospital Dr. José de Almeida, in the scope of a research protocol between ECBio–Research and Development in Biotechnology, S.A., and HPP Saúde–Parcerias Cascais, S.A. Umbilical cord donations were obtained with written informed consents according to Directive 2004/23/EC of the European Parliament (Portuguese Law 22/2007 of June 29).

### Ethics and Regulation—Animal Procedures

All animal procedures are in conformity with the Directive 2010/63/EU of the European Parliament and were approved by the IBMC-INEB (Instituto de Biologia Molecular e Celular–Instituto de Engenharia Biomédica) Animal Ethics Committee and Direcção Geral de Alimentação e Veterinária (permit 022793). Humane endpoints were followed according to the Organization for Economic Cooperation and Development Guidance Document on the Recognition, Assessment, and Use of Clinical Signs as Humane Endpoints for Experimental Animals Used in Safety Evaluation (2000).

### UCM-MSC Isolation

Human UCM-MSC were isolated according to Martins et al. (2014) and patented proprietary technology (PCT/IB2008/054067; WO 2009044379) developed by ECBio. The procedure includes three recovery phases to ensure a high cell yield and high isolation success rates. Furthermore, the UCM-MSC used in this study were obtained and processed under protocols compliant with a certifiable advanced therapy medicinal product (ATMP), compliant with clinical cell therapy. Modifications include steps for avoiding microbiological and endotoxin contamination of the final cell product, use of clinical grade enzymes, human serum as fetal bovine serum (FBS) substitute, short initial antibiotic/antimycotic decontamination in place of sustained treatment; all can be reviewed in Martins et al. (2014). Isolated UCM-MSC were cultured (up to P8) in Minimum Essential Medium α (α-MEM; Gibco, 2 mM L-glutamine, 1 g/L glucose, 2.2 g/L sodium bicarbonate), buffered with 10 mM HEPES (Gibco), hereafter designated UCM Basal Medium (BM), supplemented with 10% human serum (HS; Lonza; except otherwise stated), in a humidified incubator at 37°C, 21%O2 and 5% CO_2_. UCM-MSCs characterization procedures can be found in the Supplementary Data.

### Myocardial Infarction and UCM-MSC Delivery

Eight to twelve weeks old adult C57BL/6 mice (Charles River), independent of gender, were subjected to MI by permanent ligation of the left anterior descending (LAD) coronary artery, as previously described (Michael et al., 1995; Nascimento et al., 2014). UCM-MSC from two umbilical cord donors (UC-A and UC-B) were thawed in alpha-MEM containing 10% HS and resuspended in phosphate-buffered saline (PBS). UCM-MSC (2 × 10^5^ cells/heart) were delivered after LAD ligation by four intramyocardial injections of 5 μl each using a Hamilton syringe (30 Gauge, PST45°, 1701N, Hamilton Company). A group of control animals injected with vehicle (PBS, *n* = 6) was subjected to the same surgical procedure and post-operative care. UCM-MSC preparations were kept in ice throughout the surgical procedures and preparations older than 3–4 h were discarded. Analgesia and fluid therapy were performed by intraperitoneal (IP) injection of buprenorphine (Butador; Richter Pharma AG) and subcutaneous injection of 5% w/v Glucose Intravenous Infusion (B Braun), respectively. This procedure was repeated every 12 h up to 72 h after surgery or until full recovery.

### Transthoracic Echocardiography

Transthoracic echocardiography was performed 12 weeks after LAD coronary artery ligation and UCM-MSC delivery by using a Vevo 2100 microultrasound platform fitted with a high resolution 38 MHz microscan transducer (both from FujiFilm VisualSonics Inc.) and data analyzed by a blinded operator. Anesthesia was induced with 5% isoflurane, animals were placed on left lateral decubitus position and anesthesia maintained at 2% isoflurane throughout the procedure for data acquisition. Fractional shortening (FS) and ejection fraction (EF) were determined in parasternal long-axis (PSLAX) B-mode, using a modified Simpson's method as previously described (Sampaio-Pinto et al., 2018). Cardiac output was determined by computing stroke volume (SV) in the left ventricle outflow track (LVOT) determined using the Pulse wave (PW)-doppler mode in the subapical view, diameter of the aortic root (B-mode) and Heart Rate (HR). The Myocardial Performance Index (MPI), also known as the Tei index, was determined based on the isovolumetric contraction and relaxation times (IVCT and IVRT) and LV ejection time (LVET), all determined by PW-doppler at the mitral valve level.

### Histologic Procedures and Morphometric Analysis

At 12 weeks after surgery, hearts were collected for representative histological sampling as previously described (Valente et al., 2015). Briefly, animals were deeply anesthetized by IP injection of pentobarbital (Eutasil; CEVA, 400 mg/kg). After 4M potassium chloride (Sigma-Aldrich) injection into de left ventricle chamber, diastole-arrested hearts were harvested, briefly washed in PBS, and fixed in 10% formalin neutral buffer (Prolabo; VWR International) up to 16 h at room temperature before paraffin embedding. Representative sampling of the LV was obtained by transverse sectioning (3 μm thick) from the apex to the base of paraffin-embedded hearts with an interval of 300 μm between sections. Infarct-size assessment was performed by staining paraffin sections with modified Masson Trichrome staining (MT), according to the Trichrome (Masson) Stain kit (Sigma-Aldrich), with the following modifications: nuclei were pre-stained with Celestine Blue solution after staining with Gill's Hematoxylin and incubation for 1 h in aqueous Bouin solution to promote uniform staining. Infarcted area, infarcted midline and LV dilation were calculated using the semi-automatic MIQuant Software (Nascimento et al., 2011). LV infarcted wall thickness was determined manually using ImageJ as follows: for each section with a transmural infarction, the thickness of the wall from the epicardial to the endocardial border was measured in five equidistant points and the average for each section was determined. The results shown per heart represent the average of all infarcted sections.

### Immunofluorescence

After heat-induced epitope retrieval with Tris-EDTA buffer (95°C water bath, 35 min, ph = 9.0, Tris 1 mM and EDTA 10 mM), tissue was permeabilized with 0.2% Triton X-100 (Sigma-Aldrich) for 5 min and blocked for 1 h in 4% FBS/1% BSA in PBS. For CD31 detection, sections were incubated overnight at room temperature (RT) with goat anti-mouse CD31 (sc1506; Santa Cruz Biotechnology, Dallas, TX, USA), diluted 1:250 in the blocking solution. Thereafter, sections were incubated with AlexaFluor-568-conjugated donkey anti-goat IgG (A11057; Invitrogen) diluted 1:1000 in blocking solution for 1 h at RT and mounted using Fluoroshield containing DAPI (F6057; Sigma—Aldrich). For quantification of CD31^+^ cells, fluorescence images of stained sections were acquired with the INCell Analyzer 2000 (GE Healthcare) high-throughput microscope with a 40x dry objective (0.60 NA) and processed semi-automatically using the embedded system software. A study blinded operator established thresholds and criteria for detection and performed the analysis.

### Lentiviral Transduction of UCX®

A premade lentiviral vector encoding a cytomegalovirus (CMV) promoter-driven cassette containing the transgene for firefly luciferase (FCT005; Kerafast) was used to produce UCM-MSC lines from either donor with constitutive bioluminescence capacity, hereafter referred to as UC-A-FLuc and UC-B-FLuc. Vectors also carried a puromycin resistance gene (puro) and a woodchuck hepatitis virus post-transcriptional regulatory element (WPRE) downstream of the transgene. Transduction was performed as described in Lin et al. (2012). Briefly, UCM-MSC were cultured (since P3) in Minimum Essential Medium α with 2 mM L-Glutamine (α-MEM; Gibco) containing 20% FBS (Gibco) and 1% P/S (100 U/ml Penicillin and 100 μg/ml Streptomycin). Cells were sub-cultured at P4 (10^4^ cells/cm^2^) in six-well plates and transduction initiated after 12 h with 0.5 mL/well of complete media containing 100 μg/mL protamine sulfate (P4020; Sigma) with a multiplicity of infection of 5. After 8 h, 0.5 mL of complete media containing protamine sulfate was added to compensate for evaporation. After 24 h of initiating transduction the medium was replaced, cells allowed to recover for 48 h, and sub-cultured in complete medium containing 0.05 μg/mL puromycin up to P7 and UCM-MSC-Fluc cells were cryopreserved in FBS containing 10% DMSO. Non-transduced cells to control purification efficiency we treated with puromycin in parallel.

### Whole-Body Bioluminescence Imaging

UC-A-FLuc and UC-B-FLuc were delivered into mice hearts subjected to LAD coronary artery permanent ligation as described above. A group of animals subjected to sham surgery (*n* = 2, no ligation) was also prepared. Imaging was performed daily from day 1 to day 7, 15 min after subcutaneous injection of 3 mg D-luciferin (BT11, Biothema) in PBS (30 mg/mL). The IVIS Lumina III system was used coupled with the XGI-8 Gas Anesthesia System (both PerkinElmer) to induce anesthesia. Signal intensity analysis was performed in identical circular regions of interest centered in the thoracic cavity expressed as radiance (photons/second/cm^2^/steradian) using Living Image software (PerkinElmer) and normalized in each animal to the value read at day 1 (results presented as percentage of Day 1 for individual animals).

### Immunoblotting

Immunoblotting was performed as previously described (Vasques-Nóvoa et al., 2018). Briefly, samples were homogenized in modified RIPA buffer, proteins were separated by sodium dodecyl sulfate-polyacrylamide gel electrophoresis (SDS-PAGE) and then electroblotted onto nitrocellulose membranes (Bio-Rad). After blocking, blots were incubated with primary antibodies (Supplementary Table 1), which were subsequently detected with 700 or 800 nm infra-red dye-conjugated secondary antibodies. Protein phosphorylation status was evaluated incubating the membrane simultaneously with host mismatched primary antibodies targeting total and phosphorylated forms, which were identified with different fluorochrome-coupled secondary antibodies. Membranes were imaged by scanning at both 800 and 700 nm with Odyssey Infrared Imaging System (LICOR Biosciences). GAPDH was used as internal control.

### Hypoxia Induction

UCM-MSC seeded at 1 x 10^4^ cells/cm^2^ (37°C, 21%O_2_, 5%CO_2_) were allowed to adapt to low serum concentrations [5% human serum (HS)] until they reached 90% confluency. At this point, cells were submitted to hypoxic environment (1%O_2_; Normoxia groups kept at 21%O_2_) for 24h to mimic oxygen deprivation found upon transplantation into infarcted tissue. After one serum free wash, media was replenished with α-MEM without HS (25 mL to a 175 cm^2^ T-flask) and conditioning was carried for more 24 h. Finally, media was collected, concentrated with 3-kDa cut-off spin concentrators, and stored at−80°C until further use.

### Targeted Transcriptome Sequencing

Total RNA from UCM-MSC submitted to normoxia or hypoxia for 24 h was isolated using the RNeasy Plus Mini Kit (QIAGEN). Ion Torrent sequencing libraries were prepared according to the AmpliSeq Library prep kit protocol, and as published (Li and Zhang, 2015). RNA concentration and total RNA integrity number (RIN) were obtained using Qubit 3.0 fluorimeter and Agilent 2100 Bioanalyzer, respectively. Briefly, 10 ng of total RNA with high RIN (Average ± SD for *n* = 3 was UC-A_N = 8.87 ± 0.58, UC-A_H = 9.10 ± 0.37, UC-B_N = 8.63 ± 0.12, UC-B_H = 9.30 ± 0.41) was reverse transcribed, the resulting cDNA was amplified for 12 cycles by adding PCR Master Mix, and the AmpliSeq human transcriptome gene expression primer pool (targeting 18,574 protein-coding mRNAs and 2,228 non-coding ncRNAs, based on UCSC hg19). Amplicons were digested with the proprietary FuPa enzyme, then barcoded adapters were ligated onto the target amplicons. The library amplicons were bound to magnetic beads, and residual reaction components were washed off. Libraries were amplified, purified and individually quantified using Agilent TapeStation High Sensitivity tape. Individual libraries were diluted to a final concentration of 50 pM and pooled equally, with twelve individual samples per pool for further processing. Emulsion PCR, templating and 550 chip loading was performed with an Ion Chef Instrument (Thermo-Fisher). Sequencing was performed on an Ion S5XL^TM^ sequencer (Thermo-Fisher). Results from 3 independent conditioning experiments were analyzed on Transcriptome Analysis Console and only genes with a fold-change > ±2 and FDR *p*-value < 0.05 were considered. Heatmaps were done using the Average Linkage Clustering Method and the used distance measurement method was Spearman Rank Correlation. Gene ontology and KEGG pathways for up and downregulated terms were analyzed using Enrichr.

### *In vitro* Tubulogenesis Assay

The tubulogenesis assay was performed as described in Arnaoutova and Kleinman (2010), with slight alterations. Primary human myocardial microvascular endothelial cells (HMVEC-Cs), a potential clinical target of angiogenic mechanisms after MI, were maintained in EGM2-MV media (both from Lonza) and used at Passage 6. Matrigel growth factor reduced (10 μL, Corning) was used to coat a 15-well Angiogenesis μ-Slide (81506; Ibidi) and allowed to polymerize at 37°C for 30 min. HMVEC-C were suspended in complete media (EGM2-MV), Conditioned Media (CM) or concentrated negative control (a-MEM no cells) diluted in basal media (EBM), with a final CM concentration of 5x, and seeded at 6.5 x 10^4^ cells/cm^2^ in a total of 50 μL per well. Conditioned media from 3 independent hypoxia inductions was run in parallel, along with technical triplicate wells for each condition. After 7.5 h incubation at 37°C and 5%CO_2_ the center of each well was imaged using phase contrast microscopy on an inverted microscope Axiovert 200 (Carl Zeiss) with a 10x objective. Image analysis was performed on ImageJ (NIH) using the Angiogenesis Analyzer plugin (Carpentier, 2012).

### RT-qPCR

RNA was extracted using RNeasy Plus Mini Kit (QIAGEN) according to the manufacturer's instructions, and cDNA was synthesized using PrimeScript RT reagent kit (Takara Bio). Real-time qPCR was performed using iQ Sybr Green Supermix (Bio-Rad) and N-Cadherin specific primers preciously published (Forward 5′-AGGGGACCTTTTCCTCAAGA-3′, Reverse 5′- CAATGTCAATGGGGTTCTCC-3′) (Lee et al., 2012). Reactions were run on iCycler iQ5 Real-Time PCR system (Bio-Rad) in triplicates. Relative gene expression was normalized to GAPDH expression.

### Statistical Analysis

GraphPad Prism 8 was used for statistical analysis. Shapiro-wilk test was used to assess normality of the samples and F test or Brown-Forsythe test to probe equal variances. Datasets following a Gaussian distribution and showing same standard deviation were analyzed using independent sample Student's *t*-test and one-way ANOVA for two to three or more groups, respectively, followed by Tukey's *post-hoc* test for multiple comparisons. Statistical significance of non-parametric data was tested with Kruskal-Wallis test, followed by the FDR method of Benjamini and Hochberg adjustment for multiple comparisons.

## Results

We had previously shown that UCM-MSC attenuate remodeling after myocardial infarction upon intramyocardial delivery by proangiogenic, antiapoptotic, and endogenous cell-activation mechanisms as observed 2 weeks after MI. Herein, using the same murine MI model, the efficiency of UC-A and UC-B was evaluated in a long-term scenario of 12 weeks. UC-A and UC-B were collected from different donors using proprietary technology and updated protocols compliant with cell therapy in a clinical setting (Martins et al., 2014). Both cell lines meet the minimal criteria defined by the International Society for Cellular Therapy (Dominici et al., 2006), namely plastic cell-adherence, expression of CD73, CD105, CD90, and CD44 and absence of CD45, CD34, CD31, CD19 and HLA-DR surface markers (Supplementary Figures 1A,B, Supplementary Tables 2, 3). Despite similar MSC profile, UC-B displayed greater proliferation rates as demonstrated by higher levels of histone H3 phosphorylation (ph3) and greater metabolic activity (Supplementary Figures 1B,C). This resulted in 9.6 million cells/cm and 2.3 M/cm of cord in UC-B and UC-A at P2, respectively (data not shown).

### UCM-MSC Transplantation Consistently Improve Cardiac Function 12 Weeks After MI

**Figure 1:**
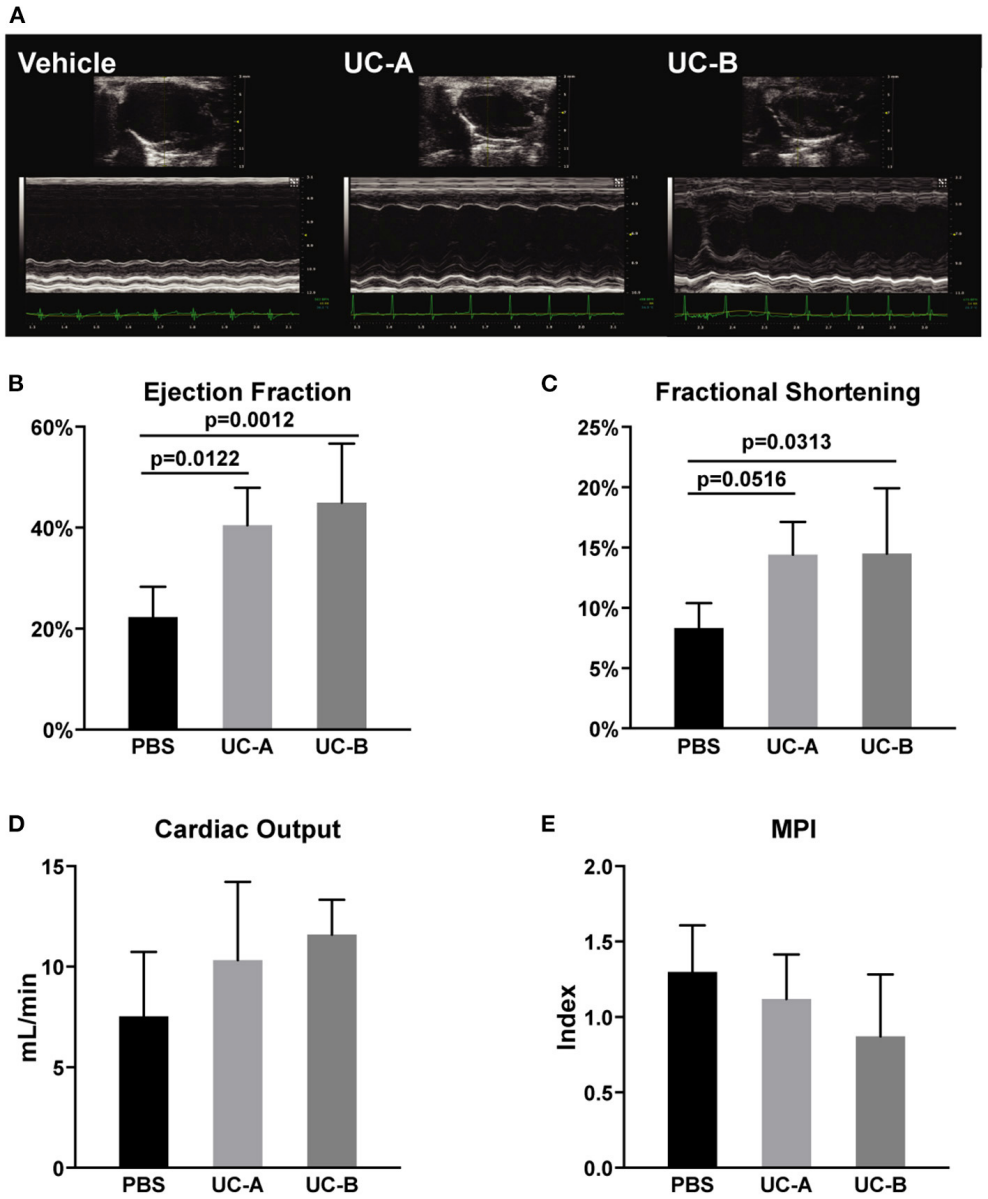
Long term improvement in left ventricle systolic function 12 weeks after MI and UCM-MSC delivery from the 2 donors. **(A)** Representative PSLAX views in B- and M-mode. **(B)** Ejection fraction and **(C)** fractional shortening calculated using the Simpson's method (PSLAX B-mode). **(D)** Cardiac output determined through the quantification of the left ventricular outflow tract stroke volume (LVOT SV) in PW-doppler mode, aortic root diameter and Heart rate. **(E)** Myocardial Performance Index was assessed computing exclusively PW-doppler data (Mitral Valve Closure to Opening Time (MVCOT) and LV Ejection time). Mean ± SD.

Cardiac function was analyzed by high-resolution echocardiography (*n* = 6 in the Vehicle Group; *n* = 5 in UC-A; *n* = 7 in UC-B). Representative images of PSLAX view of each experimental group demonstrate an attenuation of left ventricle (LV) dysfunction in the transplanted groups, when compared to the vehicle control (Figure 1A). A consistent improvement of LV functional parameters in animals treated with UCM-MSC was observed. Ejection fraction was improved from 22.3 ± 6.0% in the vehicle treated groups to 40.5 ± 7.5% in UC-A (*p* = 0.0012) and 45.0 ± 11.8% in UC-B (*p* = 0.0313) (Figure 1B). Fractional shortening was also significantly improved between the vehicle and UC-B groups (*p* = 0.0313; from vehicle treated 8.3 ± 2.1% to 14.5 ± 5.5%), although UC-A showed a similar degree of improvement (*p* = 0.0516; to 14.4 ± 2.7%) albeit not reaching statistical significance potentially due to the small animal numbers on that group (Figure 1C). An overall trend for improved cardiac output (Figure 1D) and myocardial performance index (Figure 1E) was also observed in UCM-MSC treated groups compared to control vehicle. Of note, UC-B consistently improved cardiac function compared to UC-A, which induced minor functional benefits.

### UC-B Outperforms UC-A in Reducing Adverse Cardiac Remodeling 12 Weeks After MI

**Figure 2:**
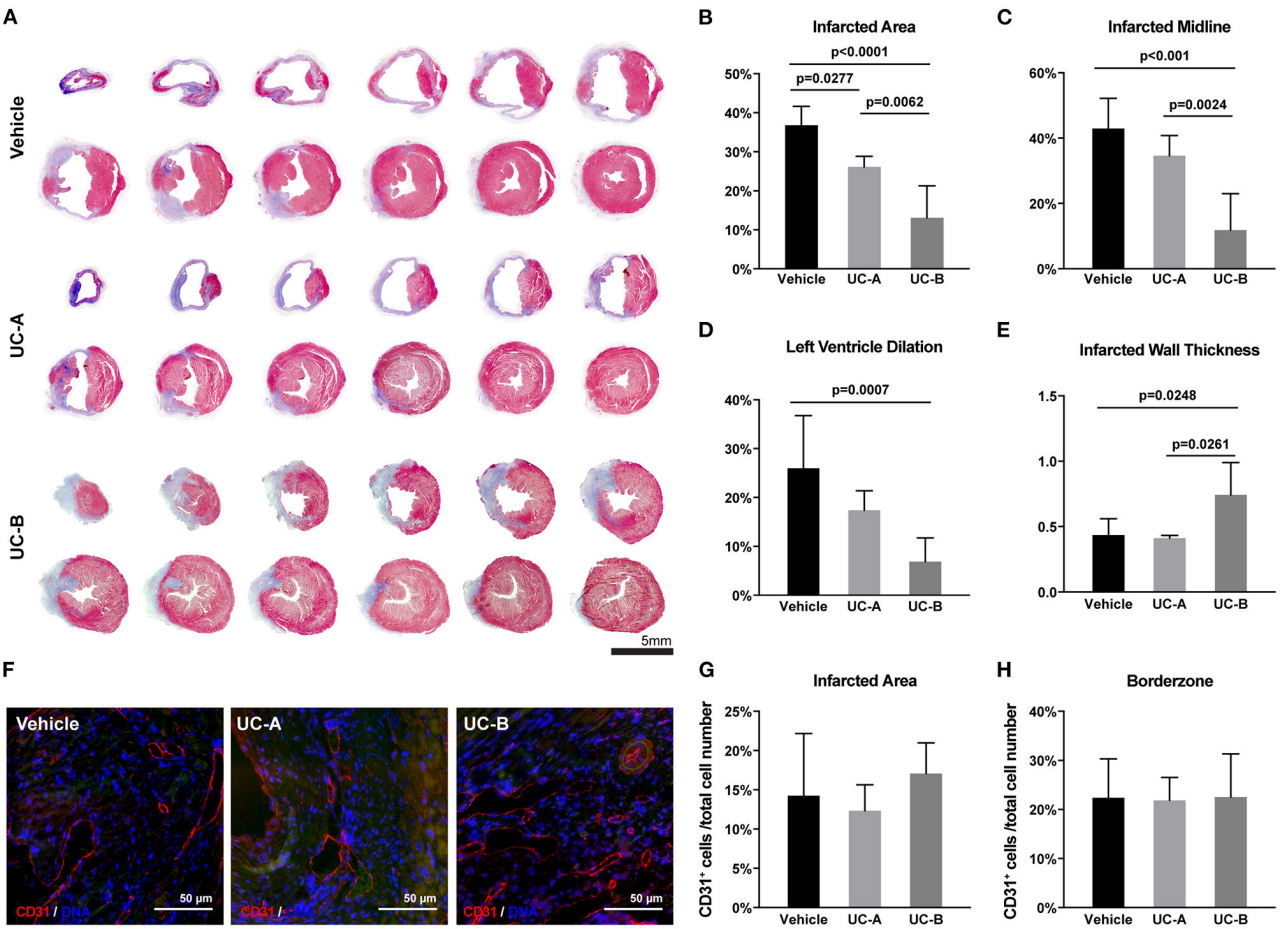
UC-B and, at less extent UC-A, decrease cardiac remodeling 12 weeks after MI. **(A)** Representative transverse histological sections of hearts stained with Masson's Trichrome of each group. Collagen deposition (scarring) is identified in blue. **(B)** Left ventricular (LV) infarcted area, **(C)** infarcted midline, and **(D)** LV lumen dilation were quantified using the *MIQuant* Software. **(E)** Infarcted wall thickness (mm). **(F)** Representative immunofluorescence images of CD31 detection in the infarcted tissue (autofluorescence in green). **(G)** Percentage of CD31-expressing cells in the infarcted area and **(H)** borderzone. Each point represents the mean of the quantification for multiple 300 μm apart sections per heart.

In line with the echocardiographic functional evaluation, a beneficial effect of UCM-MSC was observed suggesting an attenuation of MI triggered cardiac remodeling in UCM-MSC treated groups. Masson's Trichrome stained heart sections were subjected to morphometric analysis to evaluate cardiac remodeling (LV dilation and wall thickness) and infarct size was calculated in the *MIQuant* software (Figure 2A). Infarct extension reduced from 36.8 ± 4.8% in control to 26.1 ± 2.7% in UC-A (*p* = 0.0277) and to 13.1 ± 8.3% in UC-B (*p* < 0.0001). Of note, the infarct size in UC-B was smaller compared to UC-A (*p* = 0.0062) (Figure 2B). Infarct midline showed the same trend for reduction, illustrating the therapeutic efficacy of UCM-MSC, although no significant differences were found between vehicle and UC-A (from 42.9 ± 9.3% to 34.7 ± 6.2%); once more, UC-B outperformed UC-A with a midline infarct size of 11.8 ± 11.2% (*p* = 0.0024) (Figure 2C). A trend for improvement but no statistically significant differences were observed between control and UC-A treated groups (26.0 ± 10.8% vs. 17.4 ± 4.0%), while UC-B showed less dilated LV with 6.9 ± 4.8% (*p* = 0.0007 vs. vehicle) (Figure 2D). Strikingly, no differences were observed for vehicle and UC-A (0.44 ± 0.41 mm vs. 0.41 ± 0.022 mm), with UC-B performing significantly better, with an infarcted wall thickness of 0.74 ± 0.25 mm (*p* = 0.0248 vs. vehicle and *p* = 0.0261 vs. UC-A) (Figure 2E). Increased neovascularization induced by this cellular product was observed previously 2 weeks after MI in the infarct borderzone (Nascimento et al., 2014). Herein, we did not find any differences in the number of endothelial cells in the infarcted area nor the borderzone (Figures 2F–H), indicating that the distinct efficacy of the cords does not relate with increased angiogenic capacity of the tissue in this chronic phase of MI.

### UC-A and UC-B Following Myocardial Delivery Are Equally and Transiently Retained in the Myocardium

**Figure 3:**
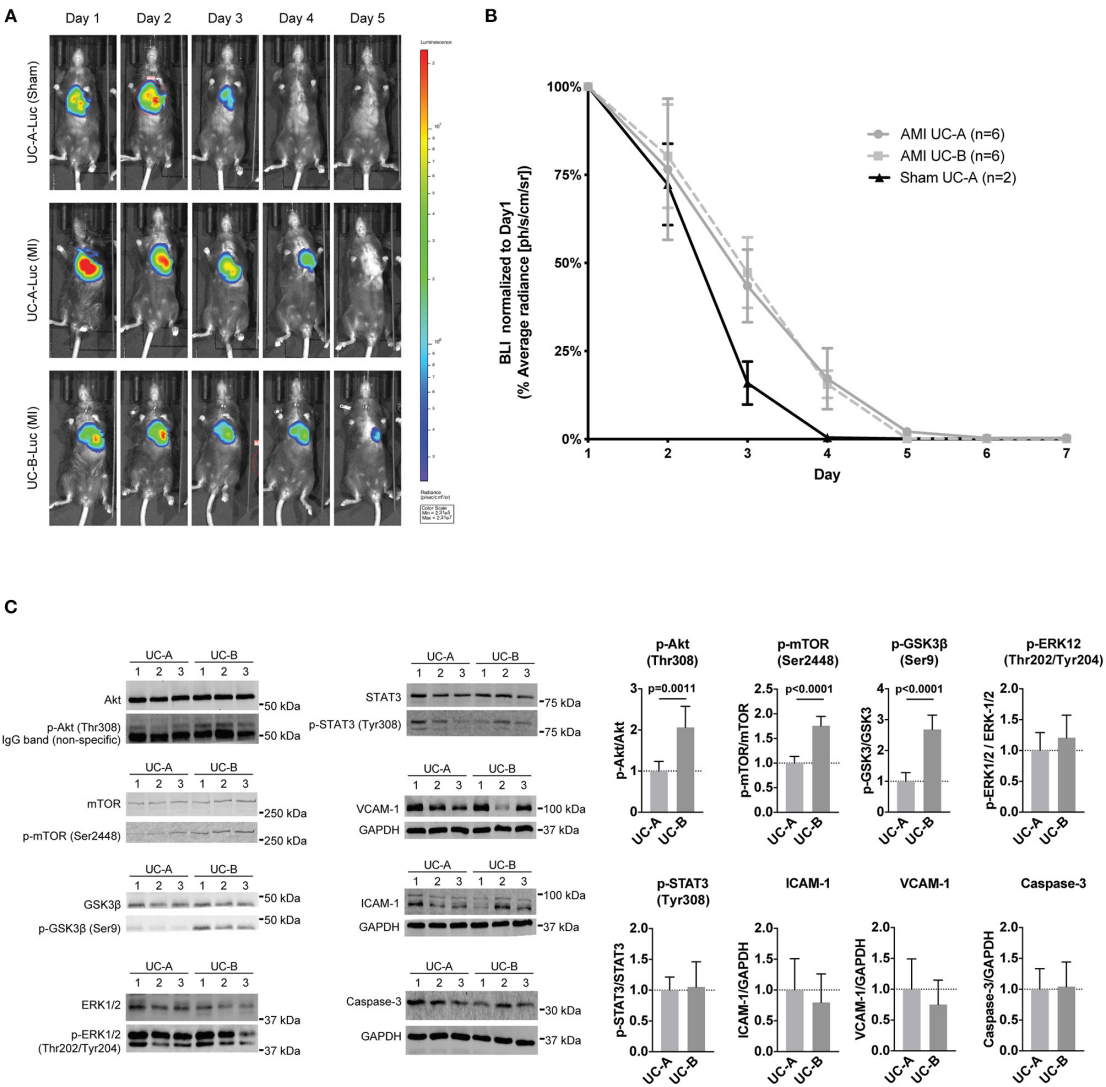
UCM-MSC delivered into the LV show similar engraftment following myocardial infarction (MI) but differentially activate signaling pathways. **(A)** Representative images of time-course bioluminescence detection of luciferase expressing UC-A and UC-B delivered into the LV tissue after MI. **(B)** Average radiance determined in the thoracic cavity area using equivalent regions of interest for all animals in the study. Data shown as Average radiance ±SEM normalized to measurements of each animal at day 1. **(C)** Western-blot analysis of the LV borderzone at day 2 post MI and UCM-MSC myocardial delivery (blots shown for 3 animals per group; *n* = 6 per study group).

We hypothesized a key factor determining different efficacy of UCM-MSC could be their ability to reside and survive for substantially different time in the infarcted left ventricle wall undergoing nutrient and oxygen deprivation and inflammation. Moreover, higher proliferation observed *in vitro* in UC-B could result in a higher number of UCM-MSCs after transplantation of similar cell numbers. To address this, UC-A and UC-B expressing firefly-luciferase under the constitutive CMV promotor after lentiviral transduction, were used in an *in vivo* longitudinal study to monitor their survival in our xenotransplant in immunocompetent infarcted mice (Figures 3A,B). UC-A-Luc and UC-B-Luc were delivered immediately upon coronary ligation in a group of 6 animals each. Upon administration of D-Luciferin at every 24 h, only viable cells carrying luciferase can produce bioluminescence. After day one, the number of cells reduced sharply and, from this point onward, ~25% decreased every day, to negligible numbers by day 5 (Figure 3B). The clearance profile observed was the same for both cords. Of note, and albeit for a small number of animals, the decrease in viable cell numbers in sham controls is steeper from day 2 to day 3 and cannot be detected by day 4, suggesting the MI environment might extend their survival in the host tissue.

### Activation of Akt Signaling in UC-B Treated Hearts Supports Improved Survival, Metabolism and Proliferation 48 h Post MI

Having established UC-A and UC-B have distinct performances *in vivo* and considering both persist for a short and similar period of time in the infarcted tissue, we assayed the short-term therapeutic potential of UC-A and UC-B after MI. For this purpose, LV borderzone was isolated 48 h after MI and UC-A and UC-B delivery (*N* = 6 per group) and main survival and inflammatory pathways were evaluated (Figure 3C). Increased Akt phosphorylation (Thr308) was observed in UC-B, suggesting higher Akt activity in UC-B treated hearts. Concurrently, GSK3β, a known repressor of metabolism, proliferation, and survival, showed significant increase in Akt-mediated inhibitory phosphorylation at Serine 9. Phosphorylation of mTOR, another downstream effector of Akt signaling, was also upregulated in the UC-B treated group suggesting increased Akt-mTOR-GSK3β signaling. STAT3 and ERK levels, pro-inflammatory and pro-remodeling pathways, showed similar activation levels in both cords. Furthermore, the expression of ICAM-1 and VCAM-1, regulated by inflammatory processes, and Caspase-3 seemed unchanged.

### UC-A and UC-B Display Comparable Transcriptomic Profiles and Adaptation to Hypoxia Stimulus

**Figure 4:**
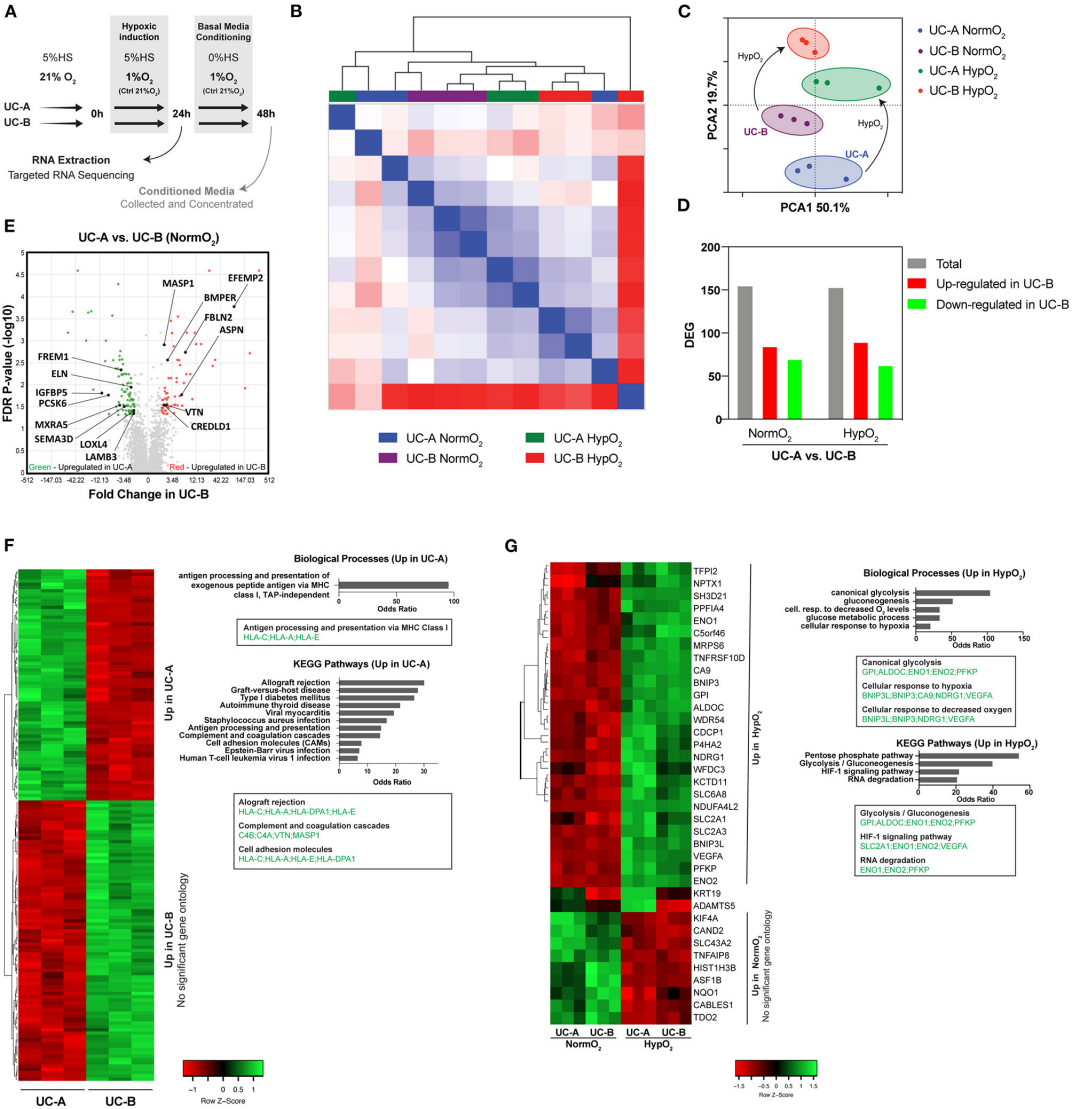
Transcriptomic profiles of UC-A and UC-B in normoxia and in response to hypoxia. **(A)** Schematic overview of the experiment. **(B)** Unsupervised hierarchical clustering of RNA-Seq datasets obtained from 3 independent experiments of each conditionUCM-MSC line. **(C)** Principal component analysis (PCA) of the 12 RNA-Seq datasets showing an overlap on PCA1 and clustering on PCA2. **(D)** Number of total differentially expressed genes (DEG) and of genes up- and down- regulated in UC-B compared to UC-A, under normoxia and hypoxia conditions (fold-change>±2, FDR < 0.05). **(E)** Volcano Plot of the DEG highlighting the statistically significant matrissome-associated genes. **(F)** Heatmap hierarchically organized by combined ranked score of DEG in normoxia conditions alongside the corresponding GO and KEGG pathways enrichment analysis. **(G)** Analysis of the DEG in normoxia vs. hypoxia for each UCM-MSC donor. Heatmap hierarchically organized by combined ranked score of the common DEG altered in the two donors in response to hypoxia; enriched GO and KEGG pathways are plotted.

Aiming at identifying gene expression differences correlating with enhanced therapeutic potential of UCM-MSC in ischemic conditions, the transcriptomic profile of the two cell-lines was compared under normoxia and hypoxic conditions, the latter mimicking environmental changes installed in MI (Figure 4A). Unsupervised hierarchical clustering of the UC-A and UC-B datasets did not show an evident association between paired experiments, neither any effect of the hypoxic treatment (Figure 4B) suggesting homogenous datasets. Moreover, principal component analysis (PCA) (Figure 4C) showed that datasets overlap on PCA1, explaining 50.1% of the sample's variance. On the PCA2 axis, which contributes 19.7% to dataset variance, both cords shift together under low oxygen levels, indicating an effect of the environmental oxygen levels, and a similar response of both UCM-MSC lines under these conditions. Regarding differential gene expression analysis (Figures 4D–F), UC-A and UC-B subjected to normoxia showed a total of 155 differentially expressed genes (DEG), of which 85 were up-regulated and 70 down-regulated in UC-B. Under hypoxia a comparable number of 153 total genes was found to be altered, with 90 upregulated and 63 downregulated in UC-B (fold change > ± 2, FDR < 0.05). Since, the pro-reparative potential of MSC in the heart has been associated with paracrine signaling (Nascimento et al., 2014), we focused our analysis on matrisome-associated proteins (including ECM-affiliated proteins, ECM regulators and secreted factors). From the reported ~1000 matrisome-associated genes (Naba et al., 2016), only 15 were differentially expressed, from which 7 and 8 were up- and down-regulated in UC-B, respectively (Figure 4E). GO enrichment analysis and pathway enrichment analysis (KEGG) of the differentially expressed genes under normoxia (Figure 4F) suggested increased activity associated with antigen presentation via MHC Class I with HLA-C, HLA-A, and HLA-E upregulated in UC-A. Pathway enrichment analysis (KEGG) supported this evidence, indicating higher transcription of genes associated with allograft rejection and cell adhesion molecules involved in inflammation (HLA-C, HLA-A, HLA-E, and HLA-DAPA1), as well as genes involved in complement and coagulation cascades (complement components, vitronectin and MASP1). Thirty seven genes showed altered expression in response to hypoxia in both UC-A and UC-B, 27 were upregulated and 9 downregulated on both cords (Figures 4B,C,G). The subset of upregulated genes showed an enrichment for processes related with cellular response to hypoxia and glycolysis; enriched KEGG pathways further hinted an adaptation of both cords to hypoxia, with enriched HIF-1 signaling pathway as well as Glycolysis and Gluconeogenesis, most notably, the upregulation of VEGF and Glucose-6-phosphate. As the two cords changed alongside in response to hypoxia, GO enrichment analysis and pathway enrichment analysis (KEGG) of the differentially expressed genes between the UC-A and UC-B after hypoxia retrieved similar results to the ones found in normoxia (data not shown). Overall, and despite similar transcriptomic profiles in normoxia and hypoxia, UC-B and UC-A expression differences were found regarding genes encoding for MHC class I molecules and complement activation-related proteins which are important elements of the inflammatory response.

### UC-A and UC-B Present Equivalent Potential to Induce Tubulogenesis in Human Cardiac Endothelial Cells *in vitro*

**Figure 5:**
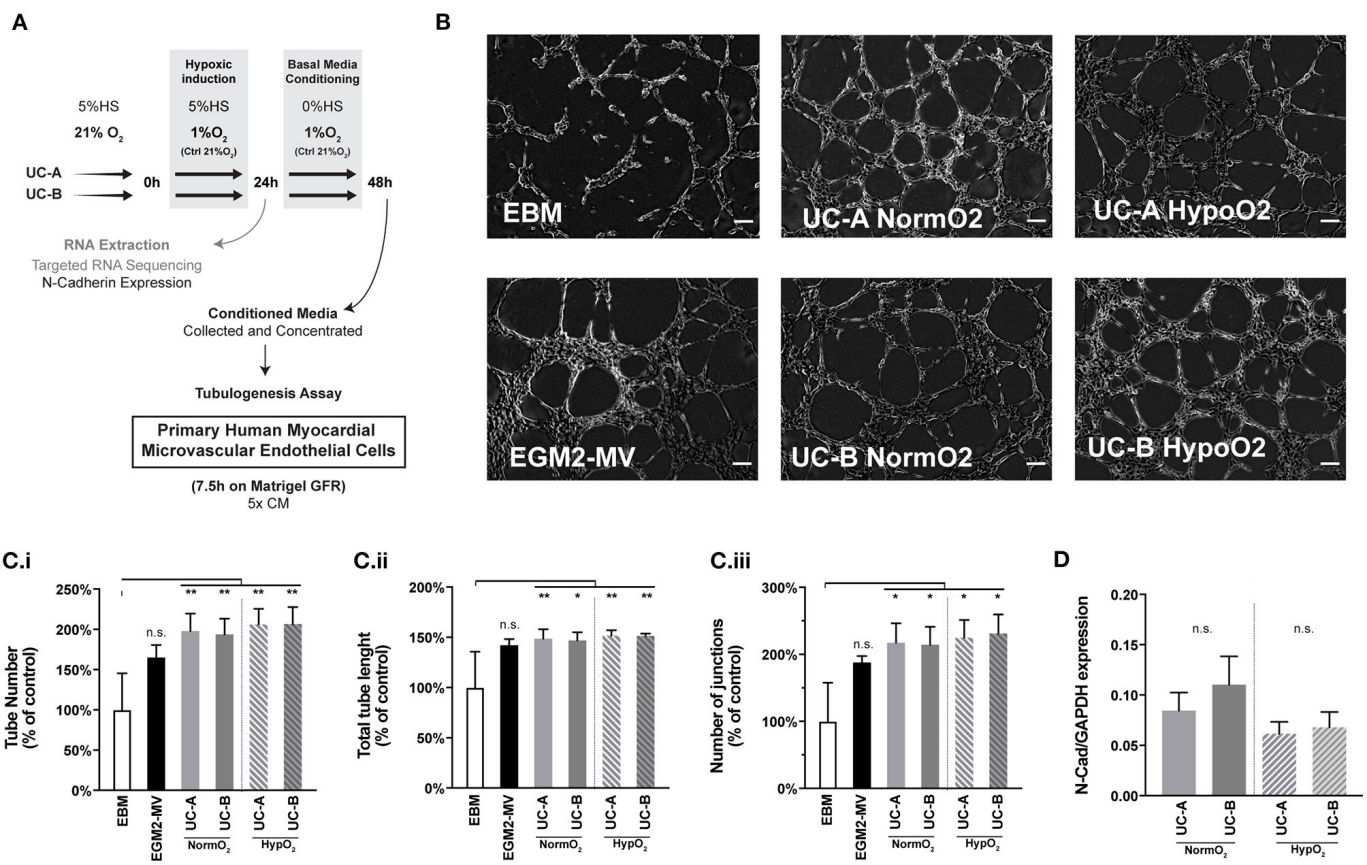
UC-A and UC-B display equivalent *in vitro* angiogenic potential on cardiac microvascular endothelial cells. **(A)** Schematic overview of the experiment. **(B)** *In vitro* tubulogenesis assay of human myocardial microvascular endothelial cells. Representative images of the formed tubular-like structures 7.5 h after incubation with the conditioned media of the 4 experimental groups. EBM (basal media) and EGM-MV (complete media) controls are shown. Scale bar: 200 μm. **(C)** (i) Total tube number. **(C)** (ii) Total Tube length. **(C)** (iii) Number of Junctions. Results of 3 independent experiments are depicted. (*p < 0.05 and **p < 0.01) **(D)** Gene expression of N-Cadherin in UCM-MSC from the two donors from 3 independent hypoxia induction experiments (*p* = 0.05). n.s. - non-significant.

We and others have previously indicated angiogenesis as one of the main mechanisms boosted by human UCM-MSC delivery upon MI (Zhang et al., 2013; Nascimento et al., 2014). As such, a classical tubulogenesis *in vitro* assay was performed to assess the angiogenic potency of the different donor-cord pairs in this study (Figures 5A–C). Human microvascular endothelial cells from cardiac origin were seeded onto a matrigel layer (growth factor reduced), in media conditioned by UC-A and UC-B and allowed to form tubes for 7.5 h. Results shown correspond to 3 independent conditioning experiments, and tubes quantified in triplicate wells for each condition/experiment. When compared to endothelial basal media (EBM), the conditioned media produced by the UCM-MSC, in either normoxia or hypoxia, increased tube number (by 98.2 ± 21.7% in UC-A-Normoxia (N), 94.0 ± 21.7% in UC-A-Hypoxia (N), 106.0 ± 19.6% in UC-B-N, and 106.5 ± 21.1% in UC-B-H), tube length (by 48.6 ± 9.38% in UC-A-N, 46.8% ± 8.15% in UC-A-H, 51.6 ± 5.58% in UC-B-N, and 51.4 ± 2.03% in UC-B-H) and branching points/junctions (by 117 ± 29.1% in UC-A-N, 114.4 ± 27.10% in UC-A-H, 125.1 ± 26.39% in UC-B-N, and 131.3 ± 28.21% in UC-B-H). Notably, no significant differences were found between the two cords nor between UCM-MSC subjected to normoxia or hypoxia environmental conditions.

### N-Cadherin Transcriptional Profile Is Identical Between Cords

It was previously suggested that transcriptional and translational levels of N-Cadherin positively correlated with the *in vivo* therapeutic efficacy of human UCB-MSCs in MI, in a study evaluating MSCs derived from a set of 4 cords (Lee et al., 2012). Based on this evidence, we assessed N-Cad expression levels on UC-A and UC-B at the end of the 3 independent conditioning experiments in an effort to predict therapeutic efficacy of our donor pair (Figure 5D). While we observe a marginal increase in the N-Cadherin levels in UC-B in normoxia, and similar trend between cords was observed in hypoxia, the differences were found to be statistically non-significant, thereby not supporting the discrepant effect observed between UC-A and UC-B.

## Discussion

Our previous research has shown that human UCM-MSC attenuate remodeling after MI in mice, albeit functional improvement was not attained likely a result of the limited time-frame (14 days) of the *in vivo* study. Whether this beneficial effect was sustained in the long term and/or was dependent of donor-to-donor variability, was not addressed. In the present study, we challenged that effect in a long-term scenario of 12 weeks and with cords derived from two different umbilical cord donors, UC-A and UC-B. Echocardiographic analysis showed that human UCM-MSC delivery in an acute phase resulted in sustained LV function, smaller infarct size and attenuated cardiac remodeling. To the best of our knowledge, this is the first evidence that transplantation of human UCM-MSC in the ischemic rodent heart provides a durable therapeutic effect at functional and histological levels. These results are consistent with the outcome reported in the preliminary work of López et al. (2013) in which functional improvement was found 32 weeks following UCM-MSC therapy. However, in this study, administered cells were isolated from rats, thus hindering translational relevance, and no systematic assessment of cardiac remodeling was performed. Another report by Hsiao et al. compared the therapeutic potential of human UCM-MSC primed or not with TGF-β2 in the context of MI. While showing attenuation of functional decline in the cell treated groups over the course of 16 weeks, no differences were shown at the endpoint vs. the control group, to support a long-term beneficial therapeutic effect (Hsiao et al., 2016).

We further show that in our model of xenotransplantation into immune competent mice, UCM-MSC transiently persisted in the infarcted myocardium for a period no longer than 5 days despite eliciting a durable therapeutic effect. Myocardial long-term engraftment of human cells has been demonstrated following delivery to immunodeficient/immunosuppressed (Chang et al., 2008; Gaebel et al., 2011; Latifpour et al., 2011; Roura et al., 2012; Im Cho et al., 2017) and immunocompetent animals (Berry et al., 2006; Zhang et al., 2013; Monguió-Tortajada et al., 2017). Of note, all these studies have assessed cellular integration in tissue sections at the experimental end-point by immunofluorescence or by using fluorescent cell tracking dyes. This contrasts with longitudinal studies in which bioluminescence has been used to trace transplanted cells. In these studies, animal derived cells transplanted into animals models showed clearance times similar to what we observed (Deuse et al., 2009; Wu et al., 2017) or at the most up to 30 days (Yan et al., 2013; Tu et al., 2019). Overall, these differences seem to reflect mostly the type of methodology used to assess engraftment. Whist *in vivo* bioluminescence is less sensitive than immunodetection to identify rare events of persistent cells, it allows reliable quantification of cell clearance/engraftment during the study. Herein, at the endpoint no human cell was detected using an anti-human antibody in histological sections (data not shown), reinforcing complete cell clearance. Moreover, differences in UCM-MSCs proliferation *in vitro* did not contribute to a higher cell number or longer survival *in vivo*. While the time for transplant clearance could be different in a human-to-human transplantation scenario, this data demonstrates that a short period of contact is sufficient for therapeutic benefits. This small time-window of engraftment is not compatible with a scenario of MSC differentiation in cardiac cells as has been demonstrated by others (Nagaya et al., 2004; Berry et al., 2006; Chang et al., 2008; Li et al., 2010). Instead, this scenario argues for the paracrine effects described for these cells in multiple reports (Nascimento et al., 2014; Yao et al., 2015; Cai et al., 2016) in which immunomodulatory properties, ECM remodeling ability and capacity to promote angiogenesis are the main mechanisms (Guo et al., 2020). Several bioengineering strategies are under development to improve retention, survival, and engraftment of transplanted cells in the myocardium (Jiang et al., 2020). Of interest, a recently developed hydrogel-based combination of UCM-MSC with endothelial cells showed that *in vitro* maturation prior to transplantation promotes vasculogenic potential and cell survival/retention after transplantation in mice (Torres et al., 2020). Although, this approach may be a valuable delivery alternative for UCM-MSCs, whether longer retention will translate in better therapeutic efficacy still requires further investigation.

While being consistently beneficial and residing in the tissue for the same period of time, we show that the extent of LV function and morphology preservation at 12 weeks exerted by UC-B was superior to UC-A, even though the cells were isolated using a proprietary protocol envisaged to produce a homogeneous product (Martins et al., 2014). Moreover, UC-B delivery resulted in increased Akt-mTOR-GSK3β signaling in the infarcted myocardium 2 days post-MI. These observations are in line with abundant evidence demonstrating that the Akt-mTOR-GSK3β pathway is an important cardioprotection mechanism by promoting cardiomyocyte survival and metabolic homeostasis (Matsui et al., 2001; Shiraishi et al., 2004; Sussman et al., 2011; Lin et al., 2015). Also, the therapeutic effect of MCS delivery to the heart has been shown to encompass the secretion of a panoply of growth factors that activate mechanisms involving PI3K/Akt/mTOR pathway (Arslan et al., 2013; Cai et al., 2016). In agreement with this perspective and our findings, exosomes released by MSC promote cardiac functional restoration and improve remodeling following delivery to the ischemic heart (Arslan et al., 2013; Kang et al., 2015). Altogether, these findings support that Akt-mTOR-GSK3β pathway is a key target for therapy in ischemic diseases (Matsui et al., 2001; Shiraishi et al., 2004; Sussman et al., 2011; Lin et al., 2015).

In retrospect, and in an effort to identify features that could justify the observed differences in therapeutic potency and capacity to activate the Akt-mTOR-GSK3β pathway, we compared the transcriptome of these cells when cultured *in vitro*. Both cellular products were considered similar in normoxia apart from higher expression of HLA-I genes in UC-A, suggesting altered antigen processing via MHC Class I and allograft rejection. MHC Class I genes are present on all nucleated cells and mediate allogeneic rejection by presenting peptide antigens to CD8^+^ T cells (Braciale, 1992), thus higher HLA-I expression on MSC following transplantation could increase the risk of rejection by the host. Yet, MSC are able to evade immune surveillance by downregulating HLA-I surface expression, even when primed with IFN-γ (Wang et al., 2019). In our study, given the high immunologic barrier to xenotransplantation, together with the hostile inflammatory milieu triggered by MI, UCM-MSC were cleared from the tissue up to 5 days post-transplant. Moreover, and regardless of having higher expression levels of MHC Class I genes, UC-A persisted in the myocardium for as long as UC-B, indicating that expression differences were not reflected on a faster clearance rate nor could justify the differential therapeutic efficacy of the two cords.

Contrasting with our previous results at 14 days after MI (Nascimento et al., 2014), hearts treated with UCM-MSC displayed a similar vascular network to the vehicle group, 12 weeks after MI. It is possible that neovascularization played a role in containing adverse remodeling and the expansion of the scar by preventing cardiomyocyte death in the border zone of the acute ischemic region and might have resolved to baseline levels at this stage. Our transcriptomic and *in vitro* angiogenesis functional analysis on human cardiac endothelial cells anticipate a similar angiogenic profile between cords, hence differences in angiogenesis may not be the cause of the observed *in vivo* variation between donors. Donor variability regarding MSCs therapeutic use have been described and linked mostly to angiogenesis. Kang et al. (2018) described a variable response to hypoxia on a set of UCB-MSCs derived from 7 cords on a panel of 4 genes (ANGPTL4, ADM, CDON, and GLUT3); better responders were associated with higher angiogenic potency *in vitro*, and showed better performance *in vivo* with 2 cords when challenged in a model of limb ischemia. Lee et al. (2012) showed different angiogenic potency that correlated with therapeutic potential of four hUCB-MSCs lines in a mouse model of MI, that could be linked to individual differences in the expression of N-cadherin, resulting in overactivated ERK that lead to increased VEGF signaling. Herein, N-cadherin nor VEGF were increased in the two cords, supporting our *in vitro* functional data regarding equivalent angiogenic induction performance. Regarding specifically MSCs derived from the UCM, Kim et al. (2019) compared the angiogenic capacity of different donor-derived UCM-MSC based of the tube forming assay and advanced four biomarkers (angiogenin, interleukin-8, monocyte chemoattractantprotein-1, and VEGF) to predict the pro-angiogenic potential of MSC *in vivo*. In our setting, hypoxia-primed cords upregulated VEGFA, but their conditioned media in normoxia vs. hypoxia showed equal potential to induce the formation of vessel-like structures by cardiac microvascular endothelial cells, meaning that VEGFA is not a key effector on its own in the angiogenic capacity of UCM-MSC.

## Conclusion

This work is, to the best of our knowledge, the first evidence that transplantation of human UCM-MSC in the ischemic rodent heart provides a durable therapeutic effect at both functional and histological levels as observed 12 weeks after MI, despite transient engraftment.

Additionally, as far as we know, this is the first report of UCM-MSC donor-related variability in the ischemic heart. However, both donors performed equally good in the tube-forming assay, and therefore none of these assays was able to predict their therapeutic *in vivo* potential. As such, and despite that angiogenesis is a key mechanism for tissue repair after MI, other assays are needed to prospectively identify the best performing MSC to be used in clinical applications. In our setting, we show that therapeutic potency may not directly link with differential angiogenic potential nor variable response to hypoxia. Instead, we hypothesize other mechanisms may be at play, such as differences in cardiac protection via Akt-mTOR-GSK3β as shown in our protein analysis 2 days after MI.

## Data Availability Statement

The datasets presented in this study can be found in online repositories. The names of the repository/repositories and accession number(s) can be found below: https://www.ebi.ac.uk/arrayexpress/, E-MTAB-9978.

## Ethics Statement

The animal study was reviewed and approved by IBMC-INEB (Instituto de Biologia Molecular e Celular–Instituto de Engenharia Biomédica) Animal Ethics Committee and Direcção Geral de Alimentação e Veterinária (permit 022793).

## Author Contributions

TL: study design, data acquisition and analysis, writing original draft. FV-N, RG, and VS-P: data acquisition and analysis, review and editing. PC, HC, JS, and RB: conceptualization, funding, review and editing. PP-d-Ȯ: conceptualization, study design, funding, supervision, review and editing. DN: conceptualization, study design, funding, supervision, data acquisition and analysis, writing original draft, review and editing. All authors contributed to the article and approved the submitted version.

## Conflict of Interest

HC and PC were shareholders of ECBio S.A. JS and RB were employees of ECBio S.A. The remaining authors declare that the research was conducted in the absence of any commercial or financial relationships that could be construed as a potential conflict of interest. The authors declare that this study received funding from ECBio S.A. The funder had the following involvement in the study: provided all cell batches required for the studies.
