# Corrigendum: Consistent long-term therapeutic efficacy of human umbilical cord matrix-derived mesenchymal stromal cells after myocardial infarction despite individual differences and transient engraftment

**DOI:** 10.3389/fcell.2023.1265005

**Published:** 2023-08-18

**Authors:** Tiago L. Laundos, Francisco Vasques-Nóvoa, Rita N. Gomes, Vasco Sampaio-Pinto, Pedro Cruz, Hélder Cruz, Jorge M. Santos, Rita N. Barcia, Perpétua Pinto-do-Ó, Diana S. Nascimento

**Affiliations:** ^1^ Instituto de Investigação e Inovação em Saúde (i3S), University of Porto, Porto, Portugal; ^2^ Instituto Nacional de Engenharia Biomédica (INEB), University of Porto, Porto, Portugal; ^3^ Instituto de Ciências Biomédicas Abel Salazar (ICBAS), University of Porto, Porto, Portugal; ^4^ Cardiovascular RandD Center, Faculty of Medicine of the University of Porto, Porto, Portugal; ^5^ Department of Internal Medicine, Centro Hospitalar Universitário São João, Porto, Portugal; ^6^ ECBio S.A., Amadora, Portugal

**Keywords:** mesenchymal stromal (or stem) cells, Wharton’s jelly, myocardial infarction, regeneration/repair, umbilical cord matrix derived mesenchymal stromal cells (hUCM-MSCs), cell therapy, donor variability, cardiac fibrosis

In the published article, there was an error in the **Funding** statement. The correct **Funding** statement appears below.

“This work was funded by the European Regional Development Fund (ERDF) through COMPETE 2020, Portugal 2020 and by FCT (Fundação para a Ciência e Tecnologia), ([POCI-01-0145-FEDER-030985 and POCI-01-0145-FEDER-016385]) and by FCT/Ministério da Ciência, Tecnologia e Inovação in the framework of individual funding [CEECINST/00091/2018] to DN and by QREN funds through the project ClinUCX (QREN 30196) and individual fellowships: [PD/BD/127997/2016] to TL, [SFRH/BD/144490/2019] to RG [SFRH/BD/111799/2015] to VS-P. The funding bodies other than ECBio had no role in design, in the collection, analysis, and interpretation of data; in the writing of the manuscript; or in the decision to submit the manuscript for publication.”

Also, the role of funder ECBio S.A. was missing from the **Conflict of interest** statement. The correct **Conflict of interest** statement appears below.

“HC and PC were shareholders of ECBio S.A. JS and RB were employees of ECBio S.A.

The remaining authors declare that the research was conducted in the absence of any commercial or financial relationships that could be construed as a potential conflict of interest.

The authors declare that this study received funding from ECBio S.A. The funder had the following involvement in the study: provided all cell batches required for the studies.”

The authors apologize for these errors and state that this does not change the scientific conclusions of the article in any way. The original article has been updated.

