# Supplementary material for: Consistent Long-Term Therapeutic Efficacy of Human Umbilical Cord Matrix-Derived Mesenchymal Stromal Cells After Myocardial Infarction Despite Individual Differences and Transient Engraftment
Source: Front Cell Dev Biol. 2021 Feb 4;9:624601. doi: 10.3389/fcell.2021.624601 (PMC7890004; doi:10.3389/fcell.2021.624601)
Supplement: Supplementary file 1 [file Data_Sheet_1.DOCX]

**Supplementary Material**

**Methods**

**Characterization of UCM-MSCs surface markers**

Flow cytometry was used to characterize UC-A and UC-B culture in P2 as described in (Santos et al., 2013), and P6. Cells were collected using a cell scraper after incubation with PBS-EDTA 5 mM (pH 7.4) during 30 min on ice with gentle agitation. The collected cell suspension was washed and subsequently stained with antibodies listed on Table 1. Cells were washed twice in FACS media (3% FBS in PBS) and analyzed on a FACS Canto II flow cytometer (BD Biosciences). Following analysis was performed on FlowJo software.

**Bright field microscopy**

Cells were seeded at 10000 cells/cm^2^ in culture media (α-MEM, 20% HS, 2 mM L-glutamine, 1% Pen/Strep) and photographed for morphology analysis using a Leica DMi1 microscope.

**Resazurin assay**

Resazurin assay was carried out in 5 consecutive days from the day after plating. Resazurin was incubated at 1:10 in culture media during 2 hours at 37°C. Fluorescence (ex: 530 nm/em: 590 nm) of samples and blank was read in triplicates in a 96-well black plate using a Synergy MX Microplate Reader (BioTek).

**Immunofluorescence**

Cells were seeded at 10000 cells/cm^2^ in an 8-well glass bottom slide (IBIDI) and fixed in 4% PFA after 24 h of culture. The cells were washed in PBS, incubated with blocking solution (1% BSA/4% FBS in PBS) for 1 h and stained for pH3 (Rabbit IgG) at 1:800 (#3377, Cell Signaling) overnight at 4ºC. Thereafter, cells were washed in PBS and stained with secondary antibody Alexa Fluor 568-conjugated donkey anti-rabbit IgG (A10042, Invitrogen) at 1:1000 for 2 h. Phalloidin CruzFluor 488 conjugate (sc-363791, Santa Cruz) was incubated for 20 min at RT after washing. Finally, DAPI was incubated at 1:1000 for 5 min at RT.

**Supplementary Figures**

**
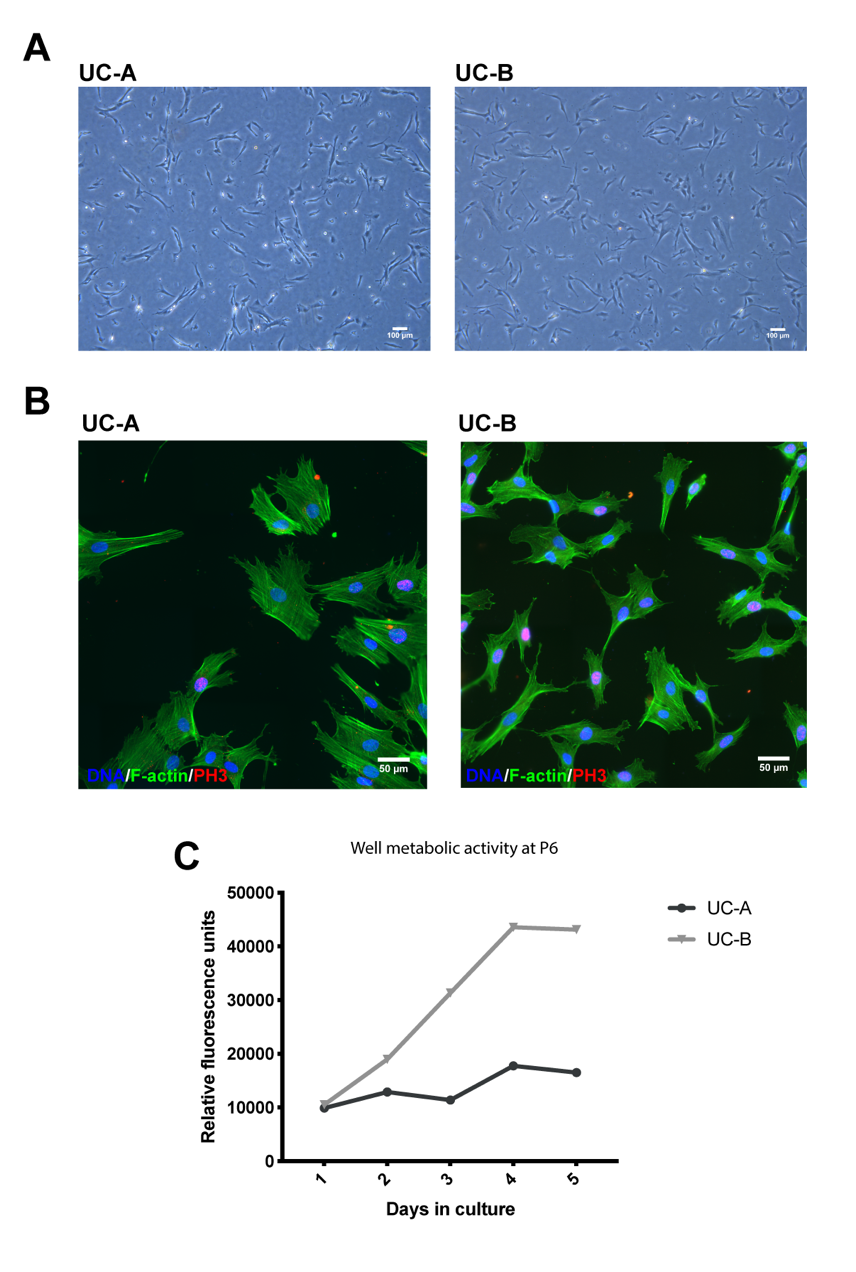
**

**Figure S1** – UCM-MSCs caracterization at P6. **A)** UC-A and UC-B display adherent fibroblast morphology the day after thawing into tissue culture treated plastic. **B)** Cytoskeleton organization is similar as shown by f-actin staining with phalloidin. Phosphohistone 3 (PH3) stained nuclei are found in both cell lines**. C)** resazurin assay performed at P6 shows increased metabolic activity in UC-B wells indicating higher cell number throughout the time course of the culture.

**Supplementary Tables**

**Table S1** – List of antibodies used for Western-blot

| **Immunoblotting** | **Isotype** | | **Source** | | **Reference** | | **Dilution** | |
| --- | --- | --- | --- | --- | --- | --- | --- | --- |
| **Primary antibodies** |  | |  | |  | |  | |
| ERK1/2 | Rabbit IgG | | Cell Signalling Technology | | 9107 | | 1:1000 | |
| p-ERK1/2 (Thr202/Tyr204) | Mouse IgG1 | | Cell Signalling Technology | | 9101 | | 1:1000 | |
| Akt | Rabbit IgG | | Cell Signalling Technology | | 9272 | | 1:1000 | |
| p-Akt (Thr308) | Mouse IgG1 | | Cell Signalling Technology | | 5106 | | 1:1000 | |
| mTOR | Mouse IgG1 | | Cell Signalling Technology | | 4517 | | 1:1000 | |
| p-mTOR (Ser2448) | Rabbit IgG | | Cell Signalling Technology | | 5536 | | 1:1000 | |
| GSK-3β | Rabbit IgG | | Cell Signalling Technology | | 9315 | | 1:1000 | |
| p-GSK3β (Ser9) | Mouse IgG1 | | Cell Signalling Technology | | 14630 | | 1:1000 | |
| Caspase-3 | Rabbit IgG | | Cell Signalling Technology | | 9662 | | 1:500 | |
| VCAM-1 | Rabbit IgG | | Abcam | | ab134047 | | 1:500 | |
| ICAM-1 | Rabbit IgG | | Sigma-Aldrich | | SAB4300383 | | 1:500 | |
| GAPDH | Mouse IgG1 | | Abcam | | ab8245 | | 1:2000 | |
| STAT3 | Rabbit IgG | | Cell Signalling Technology | | 9139 | | 1:1000 | |
| p-STAT3 (Tyr708) | Mouse IgG1 | | Cell Signalling Technology | | 9145 | | 1:1000 | |
| **Secondary antibodies** |  |  | |  | |  | |  |
| **Immunoblotting** | **Host** | **Isotype** | | **Source** | | **Reference** | | **Dilution** |
| IRDye® 800CW | Goat | Mouse IgG | | LI-COR | | 925-32210 | | 1:15000 |
| IRDye® 800CW | Goat | Rabbit IgG | | LI-COR | | 925-32211 | | 1:15000 |
| IRDye® 680LT | Goat | Mouse IgG | | LI-COR | | 925-68020 | | 1:15000 |
| IRDye® 680LT | Goat | Rabbit IgG | | LI-COR | | 925-68021 | | 1:15000 |

**Table S2** – Antibodies used for flow cytometry

| **Antibody** | **Dilution** | **Reference** |
| --- | --- | --- |
| CD73-PerCP | 1:100 | 46-0739-42, Invitrogen |
| CD105-PE | 1:25 | 21271054, ImmunoTools |
| CD90-PerCP | 1:100 | 328118, Biolegend |
| CD44-APC/Cy7 | 1:100 | 103028, Biolegend |
| CD14-APC/Cy7 | 1:100 | 367108, Biolegend |
| CD45-APC-eFLuor 780 | 1:100 | 47-0549-42 , Invitrogen |
| CD34-FITC | 1:100 | 11-0349-42, Invitrogen |
| CD31-APC/Cy7 | 1:100 | 303120, Biolegend |
| CD19-APC | 1:100 | 17-0199-42, eBioscience |
| HLA-DR-PE/Cy7 | 1:100 | 307616, Biolegend |

**Table S3** – Surface marker signature at Passage 2 and Passage 6.

|  | **UC-A** | | **UC-B** | |
| --- | --- | --- | --- | --- |
|  | **P2** | **P6** | **P2** | **P6** |
| **CD73** | 99.5% | 99.7% | 99.5% | 98.9% |
| **CD105** | 99.6% | 100.0% | 99.4% | 99.6% |
| **CD90** | 99.4% | 99.5% | 99.3% | 99.8% |
| **CD44** | 98.5% | 99.8% | 96.6% | 99.8% |
| **CD14** | 0.0% | 2.6% | 0.0% | 1.4% |
| **CD45** | 0.0% | 0.8% | 0.0% | 0.0% |
| **CD34** | 0.0% | 0.3% | 0.0% | 0.1% |
| **CD31** | 0.0% | 1.0% | 0.0% | 1.1% |
| **CD19** | 0.1% | 0.3% | 0.0% | 0.5% |
| **HLA-DR** | 0.0% | 0.1% | 0.1% | 0.7% |

**References**

Santos, J. M., Bárcia, R. N., Simões, S. I., Gaspar, M. M., Calado, S., Agua-Doce, A., et al. (2013). The role of human umbilical cord tissue-derived mesenchymal stromal cells (UCX®) in the treatment of inflammatory arthritis. J Transl Med, 11, 18. doi:10.1186/1479-5876-11-18
